# Long-term validation of a reverse transcription loop-mediated isothermal amplification (RT-LAMP) assay for the rapid detection of SARS-CoV-2 from March 2020 to October 2021 in Central Africa, Gabon

**DOI:** 10.1371/journal.pntd.0010964

**Published:** 2022-12-01

**Authors:** Haruka Abe, Yuri Ushijima, Rodrigue Bikangui, Georgelin Nguema Ondo, Ayong Moure, Yoric Yali-Assy-Oyamli, Rokusuke Yoshikawa, Bertrand Lell, Ayola A. Adegnika, Jiro Yasuda

**Affiliations:** 1 Department of Emerging Infectious Diseases, Institute of Tropical Medicine (NEKKEN), Nagasaki University, Nagasaki, Japan; 2 Centre de Recherches Médicales de Lambaréné, Lambaréné, Gabon; 3 National Research Center for the Control and Prevention of Infectious Diseases (CCPID), Nagasaki University, Nagasaki, Japan; 4 Medical University of Vienna, Vienna, Austria; 5 Institute of Tropical Medicine, University of Tübingen, Tübingen, Germany; 6 German Center for Infection Research, Tübingen, Germany; Karolinska Institutet, SWEDEN

## Abstract

**Background:**

Despite the development of several methods for diagnosing COVID-19, long-term validation of such methods remains limited. In the early phase of the COVID-19 pandemic, we developed a rapid and sensitive diagnostic method based on reverse transcription loop-mediated isothermal amplification (RT-LAMP) methodology, which is suitable for point-of-care application or for use in resource-limited settings to detect SARS-CoV-2. To assess the applicability of the RT-LAMP assay technique to resource-limited regions, such as rural areas in Africa, and to verify the usability of the method against various SARS-CoV-2 variants, the method was validated using clinical samples collected longitudinally during the pandemic.

**Methodology/Principal findings:**

First, the sensitivity of the RT-LAMP assay for detecting 10 SARS-CoV-2 variants was evaluated using viral RNA samples extracted from cell culture with a portable battery-supported device, resulting in the successful detection of 20–50 copies of the viral genome within 15 min, regardless of the variant. COVID-19 positive samples collected in Gabon between March 2020 and October 2021 were used to evaluate the sensitivity of the assay and to calculate the copy number of the SARS-CoV-2 genome. More than 292 copies of the viral genome were detected with 100% probability within 15 min in almost all tests.

**Conclusions:**

This long-term validation study clearly demonstrated the applicability of the RT-LAMP assay for the clinical diagnosis of COVID-19 in resource-limited settings of Africa, such as rural areas in Gabon. The results show the potential of the assay as a promising COVID-19 diagnostic method, especially in rural and remote regions located far from the official diagnosis facilities in urban or semi-urban areas.

## Author summary

RT-LAMP, a rapid and sensitive method for detecting pathogens, can be used in resource-limited settings. Although several RT-LAMP assays have been developed for the diagnosis of infectious diseases, reports on the long-term validation of these assays are limited. To demonstrate the applicability of the assay and to verify the usability of the method against various SARS-CoV-2 variants, it is important to conduct long-term validation studies using clinical samples collected in the region where the assay may be introduced for diagnoses. This study revealed the high sensitivity of the RT-LAMP assay for detecting viral RNAs of 10 SARS-CoV-2 variants, including the Omicron variant. Moreover, more than 292 copies of the viral genome were detected with 100% probability within 15 min using clinical samples collected between March 2020 and October 2021 in Gabon. The results demonstrate the suitability of the assay for use in rural areas of Africa where it is difficult for residents to access official COVID-19 diagnosis facilities.

## Introduction

During the past two years since the novel coronavirus disease (COVID-19) was declared a pandemic by the World Health Organization (WHO), each country has developed and implemented appropriate countermeasures suitable for controlling the spread of COVID-19. Despite the worldwide administration of vaccines, repeated waves of COVID-19 infection have been reported globally owing to the frequent emergence of novel variants of severe acute respiratory syndrome coronavirus 2 (SARS-CoV-2), which show higher infectivity and/or an ability to escape from the effects of neutralizing antibodies [1,2].

Several techniques have been developed for detecting SARS-CoV-2, such as nucleic acid amplification tests (NAATs) and lateral flow devices (LFDs). NAATs remain common methods of COVID-19 diagnosis owing to their high sensitivity and specificity [3–5]. Several reverse transcription-quantitative PCR (RT-qPCR) methods developed by research institutes worldwide have been considered gold standards [6,7]; however, RT-qPCR requires a detection instrument and stable power. Hence, the utilization of RT-qPCR in resource-limited settings or for on-site diagnosis may be difficult. LFDs are also routinely used for COVID-19 diagnosis worldwide due to their excellent usability and portability, although the lower sensitivity of LFDs than NAATs remains a potential issue [8,9]. Reverse transcription loop-mediated isothermal amplification (RT-LAMP) is a rapid and sensitive NAAT that involves a simple isothermal step and can be performed with a portable battery-driven device. It shows comparable sensitivity to RT-qPCR [10], indicating that RT-LAMP is suitable for COVID-19 molecular diagnosis, which can be completed in 15–20 min, even in settings where RT-qPCR implementation would be difficult.

COVID-19 diagnostic systems have been established in Africa, and most countries have started free vaccination programs. For gaining an appropriate understanding of COVID-19, genetic surveillance of SARS-CoV-2 is being actively conducted, with countries such as South Africa, Nigeria, and Senegal leading in surveillance. This extensive surveillance has resulted in the identification of a novel variant, Omicron, which shows high infectivity and the ability to evade the immune system [11–13]. Nonetheless, the overall pattern of spread and infectivity of COVID-19 remains unclear in Africa, especially in remote areas, possibly due to the difficulty faced by residents in accessing diagnosis facilities, which are typically set in urban or semi-urban areas. In Gabon, Central Africa, COVID-19 cases have been well-monitored since the early phase of the pandemic, and the genetic surveillance of SARS-CoV-2 is being actively conducted with continuous efforts of the government [14–17]. However, COVID-19 is diagnosed using RT-qPCR in urban or semi-urban settings in the country. Upon observation of COVID-19 symptoms in residents, diagnostic tests are difficult to perform in rural areas.

We have developed a variety of RT-LAMP assays to detect viruses that pose a concern to public health, such as Ebola and Zika viruses, for field surveillance [18–20]. Taking advantage of our experience, we developed a rapid and sensitive RT-LAMP assay to detect SARS-CoV-2, which was approved as an official diagnostic method by the government of Japan in March 2020 [21]. The RT-LAMP assay targeted the Orf1ab region and detected more than 23.7 copies of the SARS-CoV-2 genome with a 100% probability within 15 min, showing comparable sensitivity to RT-qPCR [21]. Although several other reports have been published examining the development of RT-LAMP assays against SARS-CoV-2, most assays have been evaluated using only samples derived from patients infected with the original Wuhan-type or D614G variants [22–25]. Hence, it remains unknown whether these RT-LAMP assays demonstrate similar sensitivity and specificity for the detection of other variants. Nevertheless, reports validating novel diagnostic methods are limited compared to those examining the development of these methods.

To examine the continuous usability of the RT-LAMP assay against various SARS-CoV-2 variants that have emerged since the beginning of the pandemic, we conducted a long-term validation study using clinical samples collected in Gabon, considering the future introduction of the assay to resource-limited areas. Whole-genome sequencing of SARS-CoV-2 detected in clinical samples was also attempted to accurately determine the variant, which might affect the sensitivity of the assay. As a result, the RT-LAMP assay showed high sensitivity for detecting all SARS-CoV-2 variants tested in this study. Our results demonstrate the suitability of the diagnostic assay in rural and remote areas and may help provide a precise understanding of the spread and infectivity of COVID-19 in Africa.

## Methods

### Ethics statement

This study was approved by the Institutional Ethical Committee of Centre de Recherches Médicales de Lambaréné (CERMEL) and Institute of Tropical Medicine at Nagasaki University (approval numbers CEI-007 and 170921177, respectively). Informed consent was not required as the study used anonymous samples with the total confidentiality of each sample.

### Viral RNA extracted from clinical samples and synthesis of standard RNA

Viral RNA was routinely extracted from nasopharyngeal swab samples using the QIAamp Viral RNA Mini Kit (Qiagen, Hilden, Germany) during routine COVID-19 diagnosis process at CERMEL. Viral RNAs extracted from COVID-19 positive samples were used in this study. To calculate the copy numbers of viral genomes, synthesized RNAs were prepared using the T7 RiboMAX Express Large Scale RNA Production System (Promega, Madison, WI, USA) with artificially synthesized DNA of the viral target sequence conjugated to the T7 promoter sequence, as described previously [21].

### Number of COVID-19 infection cases in Gabon

The number of new cases of infection in Gabon was obtained from the WHO COVID-19 dashboard [1]. We defined the first, second, and third waves of COVID-19 in Gabon as follows: April 27–August 17, 2020; January 11–May 31, 2021; and September 6–November 22, 2021 [17].

### Viral strains of SARS-CoV-2 variants

To obtain viral RNA, SARS-CoV-2 variants were propagated in VeroE6 cells, as previously described [26]. Culture supernatants were collected four days after infection, centrifuged at 2,000 × *g* for 15 min, and stored at −80°C until further use. The reference strain names used in this study are listed as follows: B.1.1 (D614G), hCoV-19/Japan/NGS-SC-1/2020 (GISAID accession number, EPI_ISL_481254); Alpha (B.1.1.7), hCoV-19/Japan/QK002/2020 (EPI_ISL_768526); Beta (B.1.351), hCoV-19/Japan/TY8-612-P1/2021 (EPI_ISL_1123289); Gamma (P.1), hCoV-19/Japan/TY7-503/2021 (EPI_ISL_877769); Delta (B.1.617.2), hCoV-19/Japan/TY11-927-P1/2021 (EPI_ISL_2158617); Lambda (C.37), hCoV-19/Japan/TY33-456/2021 (EPI_ISL_4204973); Mu (B.1.621.1), hCoV-19/Japan/TY26-717/2021 (EPI_ISL_4470503); Kappa (B.1.617.1), hCoV-19/Japan/TY11-330-P1/2021 (EPI_ISL_2158613); Omicron (BA.1), hCoV-19/Japan/TY38-873P0/2021 (EPI_ISL_7418017); Omicron (BA.2), hCoV-19/Japan/TY40-385/2022 (EPI_ISL_9595859). The virus strains were provided by the National Institute of Infectious Diseases, Japan, except for the hCoV-19/Japan/NGS-SC-1/2020 (B.1.1) strain, which was isolated from a clinical sample of a patient detected positive for COVID-19 at the Institute of Tropical Medicine, Nagasaki University. For the selection of BA.2 variant sequences to count mutations in the primer binding region, “complete” and “low coverage excluded” sequences submitted from Europe, Americas, Asia, Africa, and Oceania in 2022 were downloaded from GISAID (accessed on April 5, 2022). The data corresponding to 5,924 strains were obtained, and the sequences were aligned using MAFFT (https://mafft.cbrc.jp/alignment/server/). For the BA.3 variant, all “complete” and “low coverage excluded” sequences were downloaded from GISAID (accessed on April 5, 2022) containing data corresponding to 1,751 strains. After eliminating sequences outside the primer binding sites, SARS-CoV-2 strains that showed ambiguous bases or Ns in the primer binding region were removed manually.

### RT-LAMP assay

RT-LAMP assay was performed with the isothermal master mix reagent (ISO-004; Canon Medical Systems, Tochigi, Japan) using the Genelyzer FIII real-time fluorescence detection platform (Canon Medical Systems), as described previously [21]. The reaction mixture (total volume, 25 μL) contained 15 μL of isothermal master mix, 1 U of AMV reverse transcriptase (Nippon gene, Tokyo, Japan), 20 pmol (each) of FIP and BIP primers, 5 pmol (each) of F3 and B3 outer primers, 10 pmol (each) of LF and LB loop primers, and 1 μL of RNA sample (template). The primer sequences used are listed in Table 1. The reaction was performed under the following conditions: 68°C for 20 min, followed by a dissociation analysis at 95–75°C with a temperature change rate of 0.1°C/s. SARS-CoV-2 detection by RT-LAMP was performed twice for each clinical sample, and the average time to positivity (Tp) was calculated. Tp was defined as time period (minutes) from the beginning of the assay until the fluorescent signal reached 20,000. The synthesized RNAs containing the target sequence of the RT-LAMP assay were used as positive controls. Non-specific amplification was excluded by comparing the melting temperature with that of the positive control [20].

**Table 1:** Primer sequences used in RT-LAMP and RT-qPCR.

Method	Name	Type	Sequence (5′–3′)
RT-LAMP	LAMP_ORF1b_F3	F3	GGTTTTTTCACTTACATTTGTGG
	LAMP_ORF1b_B3	B3	TCCTCCAAAATATGTAATTTGCA
	LAMP_ORF1b_FIP	FIP	GCGAAGTGTCCCATGAGCTTATAAACTAGCTCTTGGAGGTTCCG
	LAMP_ORF1b_BIP	BIP	AATGCGTCATCATCTGAAGCATTTTCATAACCATCTATTTGTTCGCG
	LAMP_ORF1b_LF	LF	TCAGCATTCCAAGAATGTTCTGT
	LAMP_ORF1b_LB	LB	ATTGGATGTAATTATCTTGGCAAACC
RT-qPCR	NIID_2019-nCOV_N_F2	Fw	AAATTTTGGGGACCAGGAAC
	NIID_2019-nCOV_N_R2v3	Rv	TGGCACCTGTGTAGGTCAAC
	NIID_2019-nCOV_N_P2	Probe	ATGTCGCGCATTGGCATGGA

### RT-qPCR assay

RT-qPCR was performed using the One Step PrimeScript III RT-qPCR Mix (Takara Bio, Shiga, Japan) as reported previously [27]. The reaction mixture (total volume = 20 μL) comprised 10 μL of 2× One Step PrimeScript III RT-qPCR Mix, 0.4 μL of ROX Reference Dye, 2 μL of 10× primer-probe mixture, 1 μL of RNA sample, and 6.6 μL of RNase-free water. The primers and probes were developed by the National Institute of Infectious Diseases, Japan (NIID). Forward primer, reverse primer, and FAM-labeled probes were used at final concentrations of 500 nM, 700 nM, and 200 nM, respectively [28]. Primer and probe sequences are listed in Table 1. The RT-qPCR reaction was performed using the StepOnePlus instrument (Thermo Fisher Scientific, Waltham, MA, USA) with a thermal cycle program conducted using the following conditions: 52°C for 5 min and 95°C for 10 s, followed by 45 cycles of 95°C for 5 s, and 60°C for 35 s. A threshold cycle (Ct) value of 40 was set as the cut-off value. To quantify viral genome copies, a standard curve was generated using ten-fold serial dilutions of the synthesized standard RNA derived from RT-qPCR target sequences. Each clinical sample was analyzed using RT-qPCR to quantify the copy number of the viral genome.

### Whole-genome sequencing of SARS-CoV-2

COVID-19 positive samples assessed by RT-qPCR were subjected to whole-genome sequencing using a method similar to that reported previously [29]. Briefly, multiplex PCR was performed using primer sets designed by the online program Primal Scheme (https://primalscheme.com/) with an amplicon size of 450 bp. The template sequence was derived from the sequence of the Wuhan-Hu-1 strain (GISAID accession No. EPI_ISL_402125). Extracted viral RNA was reverse transcribed using SuperScript IV Reverse Transcriptase (Thermo Fisher Scientific) combined with random hexamers, according to the protocol of the manufacturer. Reverse-transcribed complementary DNA was used to perform multiplex PCR with Q5 High-Fidelity DNA Polymerase (New England Biolabs, Ipswich, MA, USA) as previously described [30]. Libraries were prepared using 50–500 ng of multiplex PCR products and the NEBNext Ultra II FS DNA Library Prep kit (New England Biolabs) in combination with NEBNext Multiplex Oligos for Illumina (New England Biolabs), according to the instructions of the manufacturer. After quantitative inspection of each library using the NEBNext Library Quant Kit for Illumina (New England Biolabs), we used the MiniSeq High Output Reagent Kit (Illumina, San Diego, CA, USA) to obtain complete genome sequences of SARS-CoV-2. Mapping of the paired-end reads was performed using CLC Genomics Workbench 11.0.1 software (Qiagen) using a whole-genome sequence of the Wuhan-Hu-1 strain as a template. Consensus sequences were extracted and aligned with reference strains using the BioEdit 7.0.5.3 software (http://www.mbio.ncsu.edu/BioEdit/bioedit.html). Sanger sequencing was performed to obtain complete genome sequences of several strains that showed short gaps or ambiguous sequences. After whole-genome sequencing, the lineage of each strain was analyzed using Pangolin v3.0 (https://github.com/cov-lineages/pangolin).

### Statistical analysis

Statistical data analysis, including correlation analysis, was performed using GraphPad Prism 7 (GraphPad Software, San Diego, CA, USA). The results were considered statistically significant when the *p*-value was less than 0.05.

## Results

### Collection of samples positive for COVID-19 and determination of the lineage of SARS-CoV-2 in Gabon

**Fig 1:**
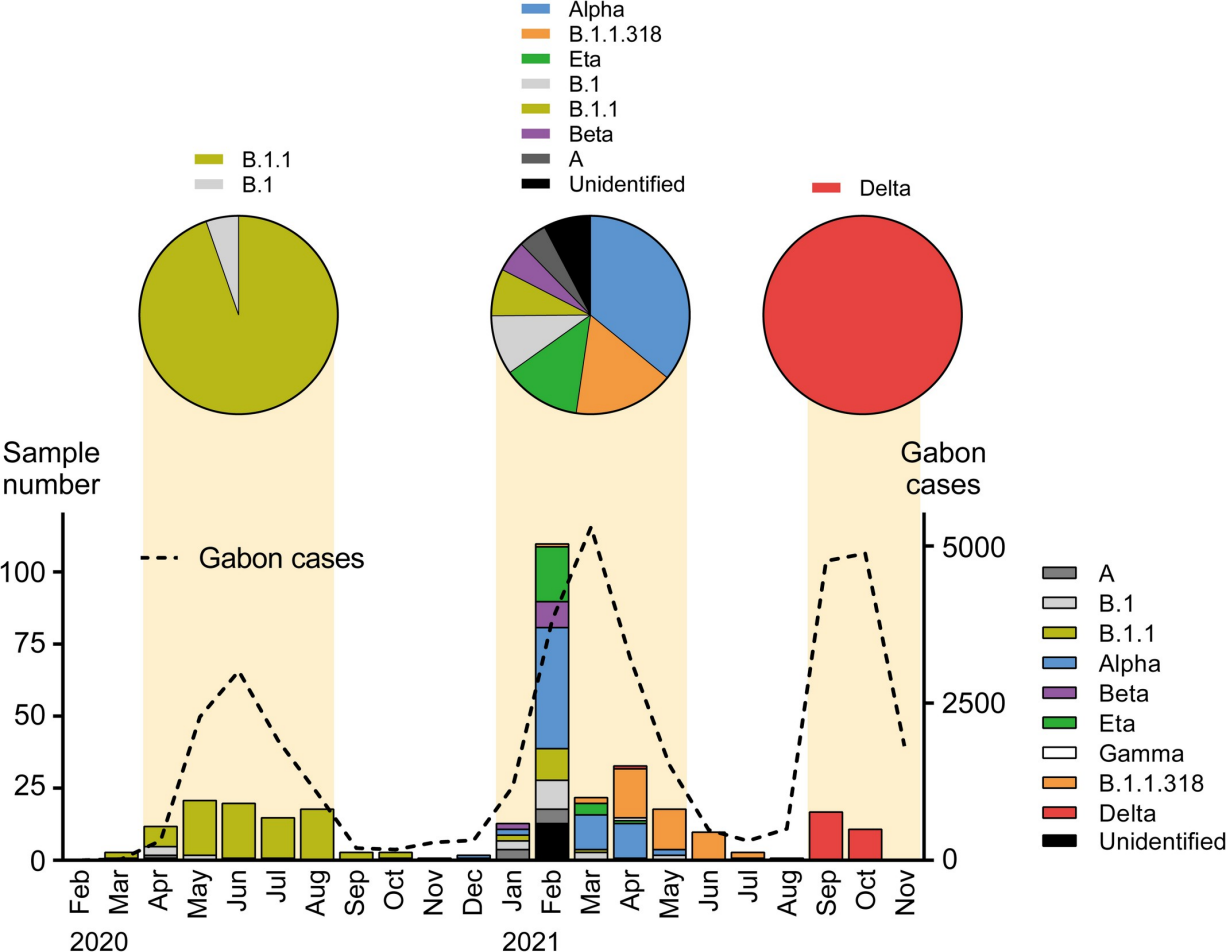
Number of collected samples and SARS-CoV-2 lineage determination during COVID-19 waves in Gabon. The number of samples collected every month is shown on the graph. Bar colors depict the lineages of SARS-CoV-2 detected in each sample. Monthly COVID-19 cases in Gabon are represented on the graph with a dotted line. The first, second, and third waves of COVID-19 are highlighted in orange, and the lineages of SARS-CoV-2 detected in samples collected during each COVID-19 wave are shown in the upper pie graph.

Continuous genetic surveillance of SARS-CoV-2 has been conducted since the beginning of the COVID-19 epidemic in Gabon in March 2020. A total of 336 samples were selected for validation of the RT-LAMP assay, including at least one sample collected every month. Although the lineages of SARS-CoV-2 were determined by whole-genome sequencing during the surveillance period [17], SARS-CoV-2 lineages of 40 samples were still unclear. We attempted to determine SARS-CoV-2 lineages in these samples using next-generation sequencing and Sanger sequencing. In total, the lineages of 310 SARS-CoV-2 strains were determined. Between March 2020 and October 2021, three major waves of COVID-19 infection were recorded in Gabon (Fig 1). The B.1.1 variant that possesses D614G substitution in the spike protein was prevalent in the first wave, and the third wave involved infection primarily by the delta variant, as observed in the corresponding wave in the African continent. A variety of variants were detected in the second wave in Gabon: B.1 variant, 9.7%; B.1.1, 7.1%; Alpha, 35.7%; Beta, 5.6%; Eta, 12.2%; B.1.1.318, 17.3%; and other minor variants, 12.2% (Fig 1 and S1 Table).

### Sensitivity of RT-LAMP assay for detection of viral RNA of SARS-CoV-2 variants

We previously developed an RT-LAMP assay using the sequence of the Wuhan-Hu-1 strain (GISAID accession number EPI_ISL_402125) as a reference [21]. To examine the sensitivity of the RT-LAMP assay for the detection of other variants of SARS-CoV-2, a dilution series of purified viral RNAs was prepared for B.1.1 (D614G), Alpha, Beta, Gamma, Delta, Kappa, Lambda, and Mu variants, corresponding to copy numbers ranging from 2 to 200,000 per reaction. The RT-LAMP assay showed a high sensitivity for all tested variants, detecting 50 copies of viral RNAs with 100% probability (Tables 2 and S2). In 20 copies of viral RNAs, the RT-LAMP assay detected B.1.1, Alpha, and Beta variants with 100% probability, whereas the detection probability of the Delta variant was 83.3%, that of Gamma, Kappa, and Mu variants was 66.7%, and that of the Lambda variant was 50.0%. The sensitivity to SARS-CoV-2 variants was consistent with previous data that showed a 100% probability of detecting 50 copies of the target RNA [21]. Particularly, the maximum average time to positivity (Tp) was 13.08 minutes (20 copies of the Lambda variant) in all tests, with Tp values lower than 15 minutes for any variant. These results indicate that the RT-LAMP assay demonstrated similar sensitivity for detecting other variants and can be considered suitable as a rapid diagnostic method for COVID-19.

**Table 2:** Evaluation of the sensitivity of RT-LAMP assay using viral RNA of SARS-CoV-2 variants.

Variant	Copies / Reaction	200000	20000	2000	200	150	100	50	20	2
B.1.1	Average Tp (min)	5.08	5.83	6.54	7.96	7.96	8.58	8.88	11.71	-
	Positivity	6/6	6/6	6/6	6/6	6/6	6/6	6/6	6/6	0/6
Alpha	Average Tp (min)	5.29	5.83	6.58	8.33	8.46	8.92	8.50	11.63	-
	Positivity	6/6	6/6	6/6	6/6	6/6	6/6	6/6	6/6	0/6
Beta	Average Tp (min)	5.29	5.83	6.50	7.46	7.29	8.00	8.42	10.21	-
	Positivity	6/6	6/6	6/6	6/6	6/6	6/6	6/6	6/6	0/6
Gamma	Average Tp (min)	5.29	5.96	6.50	7.71	7.79	8.08	7.79	9.19	-
	Positivity	6/6	6/6	6/6	6/6	6/6	6/6	6/6	4/6	0/6
Delta	Average Tp (min)	5.29	6.00	6.79	7.75	7.67	8.58	10.25	10.15	-
	Positivity	6/6	6/6	6/6	6/6	6/6	6/6	6/6	5/6	0/6
Kappa	Average Tp (min)	5.33	5.88	6.79	8.13	8.08	8.92	9.42	10.13	-
	Positivity	6/6	6/6	6/6	6/6	6/6	6/6	6/6	4/6	0/6
Lambda	Average Tp (min)	5.67	6.38	7.08	8.17	8.29	8.75	10.67	13.08	-
	Positivity	6/6	6/6	6/6	6/6	6/6	6/6	6/6	3/6	0/6
Mu	Average Tp (min)	5.29	6.00	6.67	8.00	7.92	8.92	10.58	12.25	-
	Positivity	6/6	6/6	6/6	6/6	6/6	6/6	6/6	4/6	0/6

### Validation of RT-LAMP assay using clinical samples collected between March 2020 and October 2021 in Gabon

**Fig 2:**
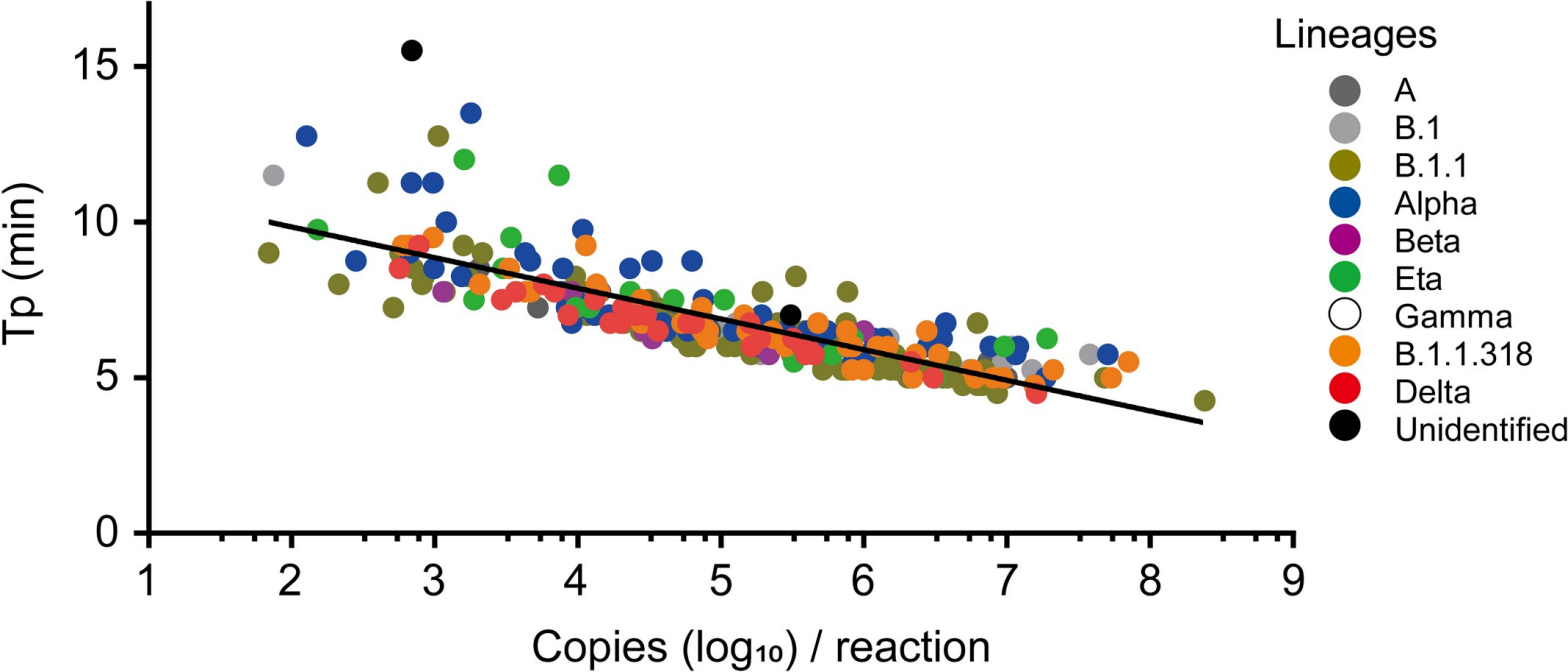
Validation of RT-LAMP assay using clinical samples collected between March 2020 and October 2021 in Gabon. The time to positivity (Tp) determined by RT-LAMP was plotted against viral genome copies per reaction for each sample assessed in this study. Colors depict the lineages of SARS-CoV-2. The correlation curve is shown on the plots.

To evaluate the accuracy of the rapid diagnostic assay, 336 clinical samples collected in Gabon were tested using the RT-LAMP assay. The results showed a significant correlation between copies per reaction and Tp (Pearson’s correlation coefficient, r = −0.809; *p* < 0.001) (Fig 2). The minimum copy number was 69.2 copies per reaction, which was detected using the RT-LAMP assay in this study. The results are consistent with those of a previous report [21]. To validate the accuracy of the RT-LAMP assay for each variant detected in this study, variant-specific detection rates were calculated (Table 3). The assay detected SARS-CoV-2 with 100% probability in samples containing more than 10^2^ copies of the Delta or B.1.1.318 variant genome per reaction. For other variants, several samples that were analyzed as positive by RT-qPCR were deemed negative by RT-LAMP for copies ranging from 10^2^–10^3^, and very few samples with <10^2^ copies were detected as positive. Overall, the RT-LAMP assay could detect SARS-CoV-2 genomes with 100% probability in clinical samples including more than 292 copies of the target. When considering the copy range of 10^2^–10^3^, the capacity of SARS-CoV-2 detection markedly decreased when using clinical samples with less than 292 copies per reaction. Several samples that were analyzed negative by RT-qPCR were included, all of which were also detected as negative by RT-LAMP, indicating no false-positive reactions using the RT-LAMP assay. This result would support the high specificity of the primers to SARS-CoV-2 as reported in our previous study that revealed no cross-reactivity of the primers with SARS-CoV [21].

**Table 3:** Validation of RT-LAMP assay using clinical samples collected in Gabon.

Variant	Copies /reaction	n	RT-LAMP					RT-qPCR	
			Positive	Negative	Sensitivity	Tp (min)	SD (Tp)	Ct value	SD (Ct)
Total	>10^7^	17	17	0	100%	5.28	0.57	19.91	1.38
(n = 336)	10^6^–10^7^	54	54	0	100%	5.45	0.53	22.98	1.04
	10^5^–10^6^	75	75	0	100%	6.20	0.59	26.30	1.02
	10^4^–10^5^	73	73	0	100%	7.05	0.69	29.64	1.11
	10^3^–10^4^	37	37	0	100%	8.53	1.56	32.94	1.24
	10^2^–10^3^	50	20	30	40%	9.64	1.93	36.87	1.20
	10^2^>	23	2	21	9%	10.25	1.77	39.38	0.86
	RT-qPCR Negative	7	0	7	-	-	-	ND	ND
A + B.1 + B.1.1	>10^7^	7	7	0	100%	5.14	0.59	20.01	1.68
(n = 134)	10^6^–10^7^	32	32	0	100%	5.30	0.46	23.08	0.97
	10^5^–10^6^	33	33	0	100%	6.18	0.71	26.68	0.99
	10^4^–10^5^	26	26	0	100%	6.79	0.43	29.90	0.91
	10^3^–10^4^	10	10	0	100%	8.65	1.56	33.57	1.24
	10^2^–10^3^	16	7	9	44%	8.61	1.28	36.87	0.93
	10^2^>	10	2	8	20%	10.25	1.77	39.49	0.88
Alpha	>10^7^	4	4	0	100%	5.63	0.43	20.54	1.02
(n = 69)	10^6^–10^7^	8	8	0	100%	6.03	0.51	23.63	1.01
	10^5^–10^6^	15	15	0	100%	6.27	0.35	26.43	0.79
	10^4^–10^5^	20	20	0	100%	7.41	0.88	29.90	1.26
	10^3^–10^4^	10	10	0	100%	8.73	1.92	32.84	1.30
	10^2^–10^3^	11	6	5	55%	10.25	1.74	36.60	1.29
	10^2^>	1	0	1	0%	ND	ND	38.64	0.00
Beta	>10^7^	-	-	-	-	-	-	-	-
(n = 9)	10^6^–10^7^	-	-	-	-	-	-	-	-
	10^5^–10^6^	3	3	0	100%	6.08	0.38	26.11	1.14
	10^4^–10^5^	2	2	0	100%	6.38	0.18	30.05	0.14
	10^3^–10^4^	2	2	0	100%	7.75	0.00	33.40	2.17
	10^2^–10^3^	1	0	1	0%	ND	ND	38.13	0.00
	10^2^>	1	0	1	0%	ND	ND	41.01	0.00
Delta	>10^7^	1	1	0	100%	4.50	0.00	19.53	0.00
(n = 29)	10^6^–10^7^	2	2	0	100%	5.25	0.35	22.33	0.39
	10^5^–10^6^	8	8	0	100%	6.09	0.33	25.67	0.66
	10^4^–10^5^	10	10	0	100%	6.90	0.29	29.09	0.87
	10^3^–10^4^	5	5	0	100%	7.60	0.38	31.77	0.67
	10^2^–10^3^	2	2	0	100%	8.88	0.53	34.90	0.33
	10^2^>	1	0	1	0%	ND	ND	39.69	0.00
B.1.1.318	>10^7^	4	4	0	100%	5.13	0.32	19.06	1.32
(n = 44)	10^6^–10^7^	10	10	0	100%	5.53	0.55	22.37	1.07
	10^5^–10^6^	11	11	0	100%	6.18	0.56	25.45	0.88
	10^4^–10^5^	12	12	0	100%	7.17	0.85	28.85	1.07
	10^3^–10^4^	4	4	0	100%	8.00	0.35	32.40	0.56
	10^2^–10^3^	3	3	0	100%	9.33	0.14	34.76	0.39
	10^2^>	-	-	-	-	-	-	-	-
Eta	>10^7^	1	1	0	100%	6.25	0.00	20.54	0.00
(n = 25)	10^6^–10^7^	2	2	0	100%	5.50	0.71	22.38	1.17
	10^5^–10^6^	4	4	0	100%	6.25	0.89	26.42	1.35
	10^4^–10^5^	3	3	0	100%	7.50	0.25	30.46	1.01
	10^3^–10^4^	6	6	0	100%	9.38	2.01	33.24	1.07
	10^2^–10^3^	5	1	4	20%	9.75	0.00	37.96	0.41
	10^2^>	4	0	4	0%	ND	ND	39.77	0.80
Not determined	>10^7^	-	-	-	-	-	-	-	-
(n = 26)	10^6^–10^7^	-	-	-	-	-	-	-	-
	10^5^–10^6^	1	1	0	100%	7.00	0.00	26.65	0.00
	10^4^–10^5^	-	-	-	-	-	-	-	-
	10^3^–10^4^	-	-	-	-	-	-	-	-
	10^2^–10^3^	12	1	11	8%	15.50	0.00	37.43	0.78
	10^2^>	6	0	6	0%	ND	ND	38.73	0.42
	RT-qPCR Negative	7	0	7	-	-	-	ND	ND

ND, not detected.

### Validation of the application of RT-LAMP assay for the detection of Omicron variants

**Fig 3:**
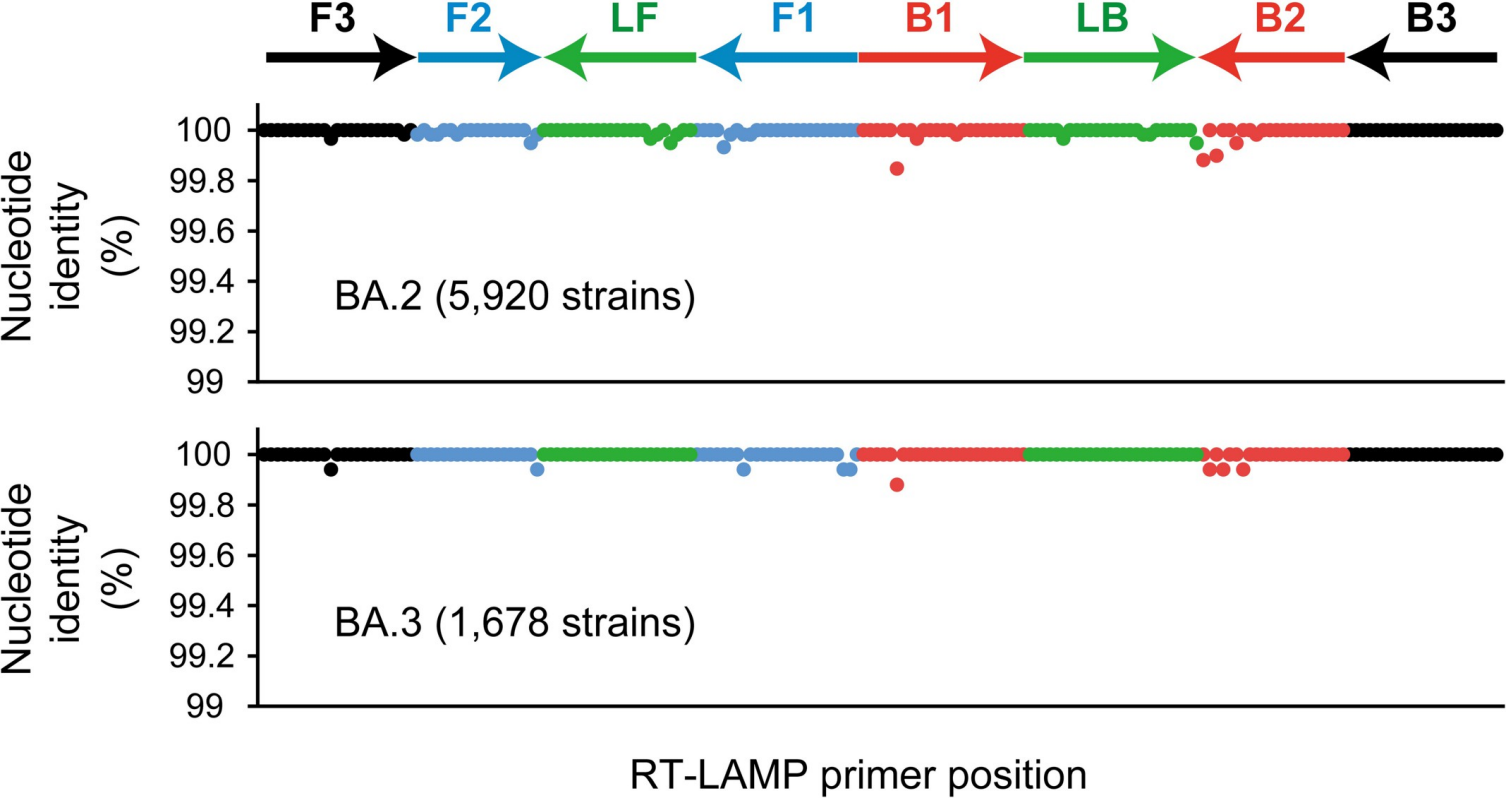
Nucleotide identity within the RT-LAMP primer binding site in BA.2 and BA.3 strains. Nucleotide identity was plotted against the nucleotide position of each RT-LAMP primer sequence. The arrows depict the length and direction of each primer. Colors indicate each primer set. A total of 5,920 and 1,678 strains were used for evaluating BA.2 and BA.3 variant sequences, respectively.

To validate the ability of the assay to detect Omicron variants, which were not included in the clinical samples assessed in this study, we evaluated the sensitivity of the RT-LAMP assay for the detection of viral RNA extracted from the culture supernatants of Omicron variants BA.1 and BA.2. The RT-LAMP assay could detect no more than 20 copies of the BA.1 genome with 100% probability, whereas it could detect 50 copies of the BA.2 genome with 100% probability and 20 copies with 83.3% probability (Table 4). The results clearly showed comparable sensitivity of the assay for the detection of Omicron and other variants. To estimate the potential ability for the detection of currently spreading BA.2 and BA.3 variants, we examined the nucleotide identity between sequences of RT-LAMP primers and Omicron variant genomes. With a selection to find “complete” and “low coverage excluded” sequences in GISAID, genomic data of 5,920 BA.2 strains submitted from regions across the globe and all deposited sequences of BA.3 strains comprising data corresponding to 1,678 strains were used in this study. Comparison between primer sequences and genomic data revealed mutations at 27 out of 186 sites in BA.2 strains (Fig 3). However, the minimum nucleotide identity was 99.85% at the sixth nucleotide of the B1 primer, and the average nucleotide identity in the entire primer binding region was more than 99.99% for BA.2 strains (Fig 3). Among 61 strains that showed mutations out of 5,920 BA.2 strains, 58 strains showed one mutation per strain, and only three strains showed two mutations in the region. Concerning the BA.3 strains, the mutations were observed at only eight sites within the primer binding region, and the minimum nucleotide identity was 99.88% (Fig 3). Among six BA.3 strains that possessed the mutation, one Indian strain (GISAID accession number, EPI_ISL_10716808) showed five mutations; however, only one mutation per strain was observed in the other five strains. As a very small population of each sublineage possessed mutations in the primer binding site, these results strongly indicated that the RT-LAMP assay would be useful for detecting Omicron variants.

**Table 4:** Evaluation of the sensitivity of RT-LAMP assay for the detection of viral RNAs of Omicron variants.

Lineage	Copies / Reaction	200000	20000	2000	200	150	100	50	20	2
BA.1	Tp (min)	5.25	6.00	6.50	6.75	7.00	7.25	8.00	7.75	-
		5.25	5.75	6.25	7.00	7.25	7.25	8.00	9.25	-
		5.25	5.75	6.50	7.00	7.00	7.50	7.50	8.25	-
		5.25	5.75	6.50	7.00	7.25	7.50	7.75	8.75	-
		5.25	6.00	6.75	7.25	7.00	7.25	7.75	7.75	-
		5.25	6.00	6.75	7.75	7.25	8.25	8.50	8.50	-
	Average	5.25	5.88	6.54	7.13	7.13	7.50	7.92	8.38	-
	Positivity	6/6	6/6	6/6	6/6	6/6	6/6	6/6	6/6	0/6
BA.2	Tp (min)	5.50	6.25	6.75	8.00	8.75	9.75	7.75	9.00	-
		5.50	6.00	7.00	8.50	8.00	10.75	10.50	9.25	-
		5.75	6.25	7.25	8.50	8.00	9.75	9.25	10.75	-
		5.50	6.25	7.00	8.75	8.75	8.00	9.75	15.25	-
		5.75	6.25	7.00	8.00	9.00	8.75	9.25	-	-
		5.50	6.25	7.00	7.50	7.75	9.25	8.50	12.00	-
	Average	5.58	6.21	7.00	8.21	8.38	9.38	9.17	11.25	-
	Positivity	6/6	6/6	6/6	6/6	6/6	6/6	6/6	5/6	0/6

## Discussion

Since the declaration of the pandemic in March 2020, systematic diagnostic methods for COVID-19 have been established in each country throughout the world. NAAT is one of the most sensitive and reliable methods for detecting SARS-CoV-2 in various types of clinical samples. Several RT-LAMP assays have been developed for COVID-19 diagnosis with the advantage of rapid and sensitive detection in a simple isothermal amplification step [22–25]. However, reports validating novel diagnostic methods are limited compared to those showing development of these methods, even though it is crucial to examine the long-term applicability of these methods.

RT-LAMP is a rapid and sensitive method for detecting target sequences using a portable detector that can be powered by a battery. Moreover, RT-LAMP is highly specific for targeted pathogens because of the use of 4–6 primers targeting 6–8 primer binding sites [31]. The advantages of the assay include diagnoses of infectious diseases on-site or in rural areas where resources and stable electricity are largely limited. Before the emergence of COVID-19, we had developed a variety of RT-LAMP assays to detect viruses that are a concern to public health, such as Ebola virus, Marburg virus, Lassa virus, and Zika virus [18–20,32–35]. In particular, RT-LAMP assays targeting Ebola and Zika viruses were tested on-site during the outbreaks, detecting target viruses within 30 min. Other groups have also actively developed several LAMP diagnostic assays for the detection of not only viruses but also other pathogens, such as malarial parasites and bacteria, for use in resource-limited settings [36–40]. Thus, RT-LAMP is recognized for its applications in on-site diagnosis or use in rural areas.

In the diagnosis of COVID-19, amplification failures, such as dropout or unusual delay, are occasionally reported with RT-qPCR. During the period when Alpha variant (B.1.1.7) was prevalent in Europe, several research groups reported S or N gene dropouts using commercial diagnostic kits based on RT-qPCR, which critically affected accurate diagnosis [41–43]. Subsequently, several cases of gene dropouts were reported in other variants, especially with S or N gene, indicating a potential risk with the use of RT-qPCR in diagnosis [44,45]. However, any point mutation in the primer binding site minimally affects amplification time and sensitivity, and is acceptable for rapid and accurate molecular diagnosis by RT-LAMP [46]. Moreover, our RT-LAMP assay could function well during the current spread of BA.2 and BA.3 Omicron variants due to very few mutations in the primer binding site (Fig 3). Fortunately, we selected the primer binding site at the nucleotide position of 21100–21355 in the orf1b region, which might show lower diversity than the S or N gene with remarkable sensitivity for use in diagnosis (Tables 2 and 4) [47]. Thus, our RT-LAMP assay demonstrated the potential for long-term usage, even in future waves of novel variants.

To date, several RT-LAMP assays have been developed to detect SARS-CoV-2, including our previous study [21–25,48,49]. Most of these assays were developed soon after the declaration of the COVID-19 pandemic and could detect 1.0 × 10^4^–2.0 × 10^1^ copies/reaction of SARS-CoV-2 genomes, indicating enough sensitivity to use in diagnosis for COVID-19. Using clinical specimens, several studies that developed novel RT-LAMP assays for COVID-19 calculated the Ct values of the detection limit by RT-qPCR, which ranged from 32 to 35 [23,26,48,49]. The detection limit of the current RT-LAMP assay was ranged within the Ct value of 33–36 using clinical specimens (Table 3), showing good consistency with previous studies. As the primer binding regions of these established RT-LAMP assays were largely distinct among each study, sensitivity and specificity seemed to be dependent simply on each primer sequence but not on specific target genes. Several studies attempted to detect SARS-CoV-2 directly from clinical specimens. Although sensitivity was 10–100-fold lower in direct RT-LAMP than that using extracted RNA samples, recent availability of reagents for the direct RT-LAMP assay may help the assay become more simple and convenient. Established RT-LAMP assays were validated using the original Wuhan-type or D614G variant, whereas a number of variants emerged after the development of these assays. Thus, it has been required to validate the established assays using newly emerged variants.

Antigen-detecting rapid diagnostic tests (Ag-RDTs) are powerful tools for clinical diagnosis at the point of care. A limit of detection (LOD) of 10^6^ genomic copies/ml (corresponding to approximately 10^2^ pfu/ml) was considered acceptable by WHO for Ag-RDTs used in clinical diagnosis [50–52], corresponding to 2.5 × 10^4^ copies/reaction in our diagnostic system (total 25 μl/reaction). The RT-LAMP assay evaluated in this study showed an LOD of 20–50 copies of targeted viral RNAs during the analysis of a variety of SARS-CoV-2 variants, and the assay detected more than 292 copies with a 100% probability in clinical diagnosis (Tables 2–4). Although there was a difference of the LOD between purified viral RNAs and clinical samples, it might be general to observe such a case due to existing inhibitors of the RT-LAMP reaction, remaining salts and ethanol, and interference of annealing or extension steps by a large amount of unnecessary specimen-derived RNAs. These results clearly show that the sensitivity of the RT-LAMP assay was high enough to detect COVID-19 infection, even if the viral titer was too low for successful detection using Ag-RDTs. Moreover, in comparison with the nucleic acid lateral-flow assays (NLFAs; e.g. dipsticks), the RT-LAMP assay shows an advantage in time to detect viruses as NLFAs usually require pre-amplification of viral genome by RT-qPCR or RT-LAMP [53]. Therefore, the RT-LAMP assay has the potential to be used as a powerful diagnostic tool at the point of care, such as Ag-RDTs.

In the African continent, four major waves of COVID-19 have been reported so far [1]. Each African country has aimed to understand the spread and infectivity of COVID-19 via systematic diagnosis and genetic surveillance that promote the submission of whole-genome sequences of SARS-CoV-2 to public databases, identifying novel variants of concern (VOCs) such as the Omicron variants [11–13]. However, more than 50% of African SARS-CoV-2 sequences were submitted from a small number of countries, including South Africa, Kenya, Nigeria, and Senegal, which are conducting extensive genetic surveillance, possibly indicating the imbalance in the capacity of diagnosis and surveillance of COVID-19 among African countries. In Central Africa, where continuous surveillance is still challenging, the promotion of the use of simple and reliable diagnostic methods is urgently needed to elucidate the spread of COVID-19 in remote areas, as it is difficult to access the diagnosis facility, which is usually set in urban or semi-urban cities. Based on this perspective, we examined the accuracy of our RT-LAMP assay in Gabon, expecting a wide distribution of the assay in rural areas of Central Africa in the future. The assay was validated for detecting a number of variants with sufficient sensitivity for COVID-19 diagnosis. It is still necessary to provide sustainable support to understand the entire situation of COVID-19 in Africa.

The limitation of the present study is the difficulty in prediction of the applicability of the RT-LAMP assay against the novel unknown SARS-CoV-2 variants that would be prevalent in the near future. RT-LAMP is more suitable for the use in resource-limited settings than other molecular diagnostic methods, whereas the assay requires several basic resources such as laboratory equipment (e.g. micropipettes and tubes) and fully-charged butteries. The procurement cost for reagents is also a critical issue to be concerned to establish a sustainable diagnostic system. The present assay was approved as an official diagnostic method in Japan, indicating the sufficient reproducibility of the result using the assay. Further validation of the reproducibility by local researchers/clinicians will strengthen the advantage of the assay. For the worldwide use of the current assay including the resource-limited regions, cooperation must be required among researchers, companies, international public health organizations, and the government of each country to appropriately overcome those challenges.

## Supporting information

S1 Table Number of collected samples and SARS-CoV-2 lineage determination during COVID-19 waves in Gabon. (XLSX)

S2 Table Evaluation of the sensitivity of RT-LAMP assay using viral RNA of SARS-CoV-2 variants. (XLSX)
